# Advances in Multi-Modality Imaging in Hypertrophic Cardiomyopathy

**DOI:** 10.3390/jcm13030842

**Published:** 2024-02-01

**Authors:** Fraser C. Goldie, Matthew M. Y. Lee, Caroline J. Coats, Sabrina Nordin

**Affiliations:** 1 School of Cardiovascular & Metabolic Health, University of Glasgow, Glasgow G12 8TA, UK; fraser.goldie@glasgow.ac.uk (F.C.G.); matthew.lee.2@glasgow.ac.uk (M.M.Y.L.); caroline.coats@glasgow.ac.uk (C.J.C.); 2 Department of Cardiology, Queen Elizabeth University Hospital, Glasgow G51 4TF, UK

**Keywords:** hypertrophic cardiomyopathy, multi-modality imaging, echocardiography, cardiac magnetic resonance imaging, cardiac computed tomography, nuclear imaging

## Abstract

Hypertrophic cardiomyopathy (HCM) is characterized by abnormal growth of the myocardium with myofilament disarray and myocardial hyper-contractility, leading to left ventricular hypertrophy and fibrosis. Where culprit genes are identified, they typically relate to cardiomyocyte sarcomere structure and function. Multi-modality imaging plays a crucial role in the diagnosis, monitoring, and risk stratification of HCM, as well as in screening those at risk. Following the recent publication of the first European Society of Cardiology (ESC) cardiomyopathy guidelines, we build on previous reviews and explore the roles of electrocardiography, echocardiography, cardiac magnetic resonance (CMR), cardiac computed tomography (CT), and nuclear imaging. We examine each modality’s strengths along with their limitations in turn, and discuss how they can be used in isolation, or in combination, to facilitate a personalized approach to patient care, as well as providing key information and robust safety and efficacy evidence within new areas of research.

## 1. Background

Hypertrophic cardiomyopathy (HCM) is characterized by abnormal growth of the ventricular myocardium and the identified culprit genes mainly relate to cardiomyocyte sarcomere structure and function [1]. The recently published European Society Cardiology (ESC) cardiomyopathy guidelines’ [2] diagnostic criteria for HCM are summarized in Table 1.

Historically, HCM was thought to be a disease of left ventricular outflow tract obstruction (LVOTO) [3] but as knowledge of the molecular basis of the disease has grown, and imaging techniques evolved, we now appreciate that it is a disease of myocyte hypercontractility and myofilament disarray associated with myocardial fibrosis, microvascular dysfunction, and left ventricular hypertrophy (LVH) [4]. The clinical consequences include adverse left ventricular remodelling, heart failure (HF), arrhythmias, and sudden cardiac death (SCD) [5,6]. The prevalence of HCM is 2–5 per 1000 of the general population [7].

**Figure 1:**
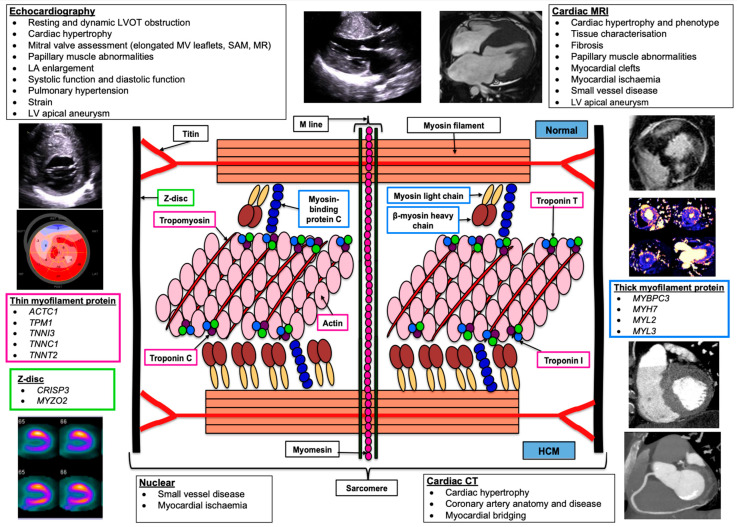
Cardiac sarcomere components and corresponding genes, and main imaging modalities with examples of their utility. ACTC1: α actin, CRISP3: Cysteine−rich secretory protein 3, HCM: Hypertrophic cardiomyopathy, MYBPC3: Cardiac myosin−binding protein C3, MYH7: Myosin heavy chain 7, MYL2: Myosin light chain−2, MYL3: Myosin light chain3, MYZO2: Myozenin 2 (calsarcin 1), TNNC1: Troponin C1, TNNI3: Troponin I3, TNNT2: Troponin T2, TPM1: Tropomyosin 1.

In up to 50% of patients, HCM is inherited as a Mendelian genetic trait and in those who undergo genetic testing, ~40–60% are found to have a single rare gene variant identified [8]. These variants are most commonly found in the sarcomeric protein genes β-cardiac myosin (*MYH7*) and cardiac myosin-binding protein C (*MYBPC3*) [9]. Figure 1 illustrates the components of a cardiac sarcomere and the relevance in HCM. Variants in *TNNT2* (cardiac troponin T), *TNNI3* (cardiac troponin I), and *TPM1* (α-tropomyosin) are less common causes of HCM and together are responsible for <10% of cases. Even less frequently, variants are identified in *ACTC1* (cardiac α-actin), *MYL2* (myosin light chain 2), *MYL3* (myosin light chain 3), and *CSRP3* (cysteine and glycine-rich protein 3). Proposed molecular mechanisms for explaining the hyper-contractile phenomenon include alterations in the actin-activated β-cardiac myosin chemo-mechanical ATPase cycle, an increased number of functionally accessible myosin heads (i.e., decrease in the super-relaxed state of myosin), and alterations in load dependence contractility that changes the power output of cardiac contraction [10,11]. In this article, we will build on previous reviews [12,13,14,15], published prior to the publication of the ESC cardiomyopathy guidelines, and will outline the role of multi-modality imaging (MMI) in the clinical pathway of HCM. We will explore each modality in turn and discuss their strengths and weaknesses and how an MMI approach provides a holistic and comprehensive assessment in HCM.

## 2. Role of Multi-Modality Imaging in HCM

### 2.1. Diagnosis and Classification

Due to an increased use of genetic testing, many patients are now seen within clinical services with an earlier phenotype of HCM. MMI plays an important role in accurate disease staging, particularly in the pre-clinical stage, which is recognized by ECG abnormalities, subtle echocardiographic abnormalities (e.g., impaired left ventricular (LV) relaxation, mitral valve abnormalities, or subtle left atrial (LA) dilatation), raised biomarker levels (type I collagen precursors), and evidence of coronary microvascular dysfunction [16]. A well-phenotyped population is essential for clinical practice and research, particularly the pre-clinical phenotype, as more mechanistically targeted therapies are developed [17,18]. The 2022 American Heart Association (AHA)/American College of Cardiology (ACC) guidelines [19] define heart failure stages with reference to signs and symptoms and heart function. With reference to this staging in the context of genetic cardiomyopathy, we propose that stage B should be separated into stages B1 and B2 to better define those who are asymptomatic with pre-clinical disease (Stage B1) from those who are asymptomatic with definite cardiomyopathy (Stage B2). The pre-clinical phenotype could be further sub-categorized into electrical and structural pre-clinical phenotypes rather than just the defined LVH diagnosis. It would enable a more nuanced delineation of these asymptomatic patients from Stage A patients, i.e., gene-positive patients who are phenotype-negative.

Once a clinical diagnosis of HCM is confirmed, MMI is essential in treatment decision making, including pharmacological and device therapy. HCM is classified as either obstructive HCM (oHCM) (characterized by dynamic LVOTO) or non-obstructive HCM (nHCM) (characterized by the absence of significant LVOTO (i.e., <30 mmHg) at rest or with provocation). Transthoracic echocardiography (TTE) is used to classify HCM and allows monitoring for change in the LVOT gradient and left ventricular ejection fraction (LVEF) and guides therapy. It is essential that imaging techniques can accurately identify issues and are also practical so that regular follow up of a large number of patients is feasible. Resting and stress imaging provide valuable information to decipher the mechanisms behind symptoms, helping to guide timely and effective treatment strategies.

Cardiac magnetic resonance (CMR) imaging is helpful at the diagnosis stage to exclude conditions that can mimic HCM on echo, for example, amyloid, hypertensive heart disease and rare conditions like Fabry disease.

**Figure 2:**
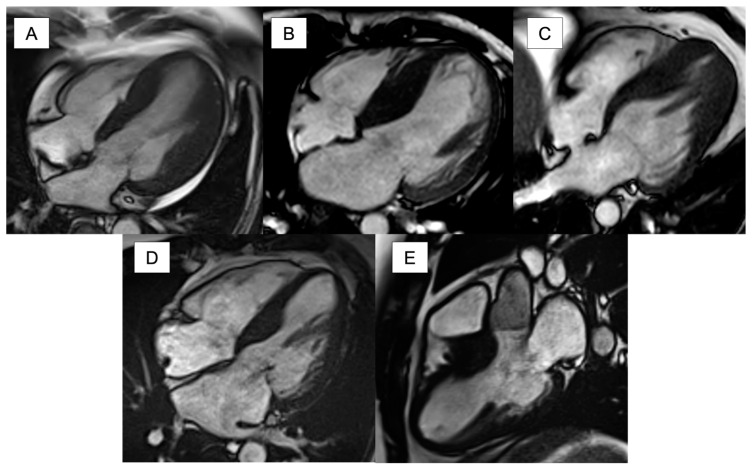
Patterns of left ventricular hypertrophy in hypertrophic cardiomyopathy with cardiovascular magnetic resonance. (**A**) Neutral pattern; (**B**) reverse curve hypertrophy pattern; (**C**) apical hypertrophy pattern; (**D**) 4-chamber view of basal septal hypertrophy pattern; (**E**) 3-chamber view of basal septal hypertrophy pattern.

Several patterns of HCM have been described including asymmetric basal septal (often referred to as ‘classical’ HCM), reverse septal, neutral, concentric, and apical (Figure 2). There appears to be differences in imaging findings depending on the pattern; for example, late gadolinium enhancement (LGE) on CMR is more common with the reverse septal pattern than the basal septal pattern [20,21].

### 2.2. Decision Making

#### 2.2.1. Pharmacological

Initial therapy for oHCM has focused on lowering LVOTO with negative inotropic agents [7]. Until recently, therapy for HCM has been focused on symptom relief with no specific targeted therapies and few randomized controlled trials. Nevertheless, imaging plays a key role in eligibility/selection, dose titration, and monitoring.

Non-dihydropyridine calcium channel blockers, β-blockers, and disopyramide all have different mechanisms of action, but their mutual negative inotropic effects improve diastolic filling time and reduce LVOTO, to alleviate symptoms [8]: despite this, none have impacted on prognoses or rates of sudden death [22]. Recently, studies have investigated the role of a new class of medication called cardiac myosin inhibitors (CMIs) (i.e., mavacamten and aficamten), and further trials are being undertaken [23,24,25,26,27,28]. Echocardiographic monitoring of LVEF is recommended in those with oHCM treated with mavacamten because of the risk of developing LV systolic dysfunction.

Surgical myectomy and septal ablation decrease LVOTO and symptoms but confer greater risks than drug therapy given their invasive nature [29], and as we will outline, imaging is essential in making decisions around the suitability and timing of intervention, as well as the actual planning and undertaking of these procedures.

#### 2.2.2. Risk Stratification

Decisions around the prevention of sudden cardiac death with implantable cardiac defibrillator (ICD) implantation can be challenging. Various risk stratification tools have been developed to guide these decisions, and all these tools require imaging data as part of their scoring algorithm.

The European HCM risk SCD calculator incorporates maximal wall thickness (MWT), LA diameter, maximal LVOT gradient (rest/Valsalva provoked), family history of SCD, non-sustained ventricular tachycardia (NSVT), unexplained syncope, and age [30]. The 2020 AHA/ACC HCM guidelines include additional risk factors of LVEF ≤ 50%, apical aneurysm, or extensive fibrosis (i.e., diffuse and extensive LGE on CMR imaging) [31].

Notably, the HCM risk SCD calculator was not validated for use after septal reduction therapy (SRT) and does not allow for the incorporation of additional risk factors, for example, those outlined in the AHA HCM SCD calculator [30,32].

#### 2.2.3. Screening

The ESC cardiomyopathy guidelines [2] indicate the importance of MMI in those at genetic risk of HCM. They advise that all first-degree relatives of patients with cardiomyopathy should be offered clinical screening with ECG and cardiac imaging (echocardiogram and/or CMR). Lorenzini et al. highlight the value of CMR in screening gene carriers [33].

## 3. Electrocardiography

While not strictly an imaging modality, electrocardiography (ECG) remains an invaluable investigation in the diagnosis and follow up of structural heart disease, particularly HCM. Most patients with HCM have abnormal ECG findings, which are non-specific and often represent the reason for referral of asymptomatic patients undergoing sports, professional, or family screening [34]. In family screening, ECG has proven to be more sensitive than echocardiography, indicating that the electric phenotype may precede morphological manifestations of disease, i.e., a pre-clinical electrical phenotype [35,36]. Common ECG abnormalities in HCM include signs of LV strain and hypertrophy, deep Q waves (particularly in the inferior and lateral leads), diffuse T wave inversion, *p* wave prolongation, intraventricular conduction abnormalities, and QTc prolongation [34].

It is well established that patients with HCM are predisposed to arrhythmia, which may include virtually all known rhythm disturbances [34]. Electrocardiography (12-lead ECG, ambulatory ECG, implantable loop recorders) can identify conduction disorders, arrhythmias, repolarization abnormalities, and LVH voltage criteria.

Patients without ECG abnormalities are associated with a less severe phenotype and more benign outcome compared to the overall HCM population [37]. In addition to the utility for risk stratification in known disease, there is evidence that ECG abnormalities may be evident in the absence of LVH on an echocardiogram, which means ECG remains vital [34].

Research into novel techniques to further utilize ECG information is ongoing. Electrocardiographic imaging (ECGI) reconstructs the electrical activity of the heart and produces reconstructions of the activation and recovery sequence of the heart, premature beats or tachycardia origin, re-entrant arrhythmias, and other electrophysiological quantities of interest. These can be reconstructed in a digitized model of the patient’s three-dimensional heart, and could enable more personalized care [38], especially in those with HCM. Webber et al. have proposed the integration of CMR imaging data with CMR-ECGI and have proven both feasibility and good reproducibility and could provide novel insights into arrhythmogenesis to enable further personalized risk stratification [39].

## 4. Echocardiography

Two-dimensional (2D) TTE is the initial and preferred imaging modality in HCM. TTE is relatively inexpensive and widely available and recommended for all patients at initial evaluation [2].

It has utility in diagnoses, monitoring, screening, and prognostication. As shown in Figure 1, TTE enables assessing the severity and pattern of LVH, LVOTO, and mitral valve pathology, as well as both systolic and diastolic function [13]. TTE plays an important role in distinguishing between oHCM and nHCM.

### 4.1. Left Ventricular Hypertrophy

TTE is recommended in the evaluation of LV wall thickness and the determination of which LVH pattern of HCM is present. The ESC guidelines outline that this should be carried out by examining LV segments ‘from base to apex examined in end-diastole, preferably in the 2D short-axis view, ensuring that the wall thickness is recorded at mitral, mid-LV, and apical levels’ [2]. When TTE image quality is suboptimal, contrast can be used to enhance information and is particularly useful in the detection of apical hypertrophy. While useful, it is important to appreciate the potential risk of interobserver variability in wall thickness measurement with TTE [40]. It is important to recognize the differences in wall thickness and cardiac dimensions between men and women in a normal population and St. Pierre et al. propose that as such, diagnostic criteria for cardiomyopathies should be more sex-specific [41]. Huurman et al. demonstrated that in genotype-positive patients referred for family screening, differences in MWT across gender are mitigated after indexation by (body surface area) BSA or weight [42].

### 4.2. Left Ventricular Outflow Tract Obstruction

**Figure 3:**
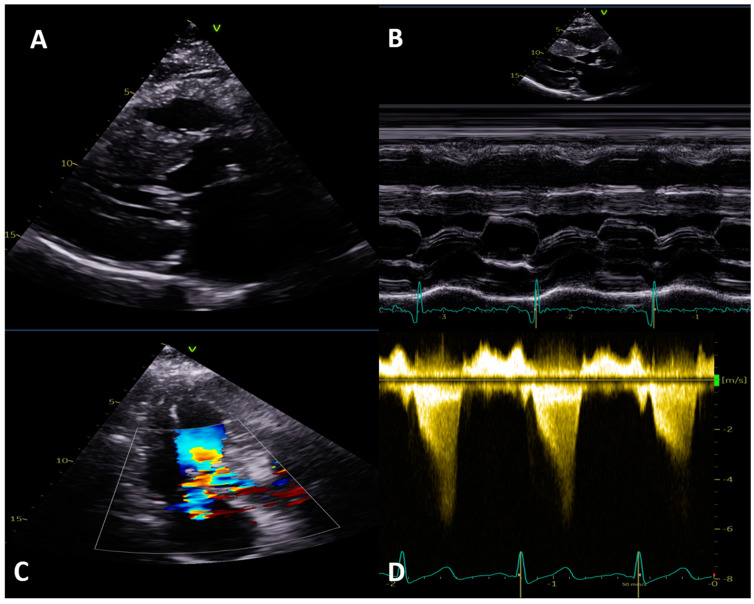
Left ventricular outflow tract obstruction (LVOT) in hypertrophic cardiomyopathy with transthoracic echocardiogram. (**A**,**B**) Systolic anterior movement of anterior mitral valve leaflet on parasternal long axis and M-mode. (**C**) Colour flow showing LVOT acceleration with associated posteriorly directed mitral regurgitation. (**D**) Continuous wave Doppler showing typical ‘dagger shape’ Doppler tracing indicating LVOT obstruction.

LVOTO occurs in up to two-thirds of patients with HCM either at rest or only on provocation [13]. LVOTO is a dynamic phenomenon and can vary even within the same patient and is dependent on afterload, preload, and LV contractility. Evidence of LVOTO is seen on M-mode, colour Doppler, and pulsed (PW) and continuous wave (CW) Doppler (Figure 3). TTE colour Doppler is used to localize turbulent flow or for aliasing, which indicates increased velocity; then, CW and PW Doppler in the apical 5- or 3-chamber views can be used to precisely localize and quantify any obstruction [13]. In all patients undergoing rest TTE, a Valsalva manoeuvre should be performed: however, due to patients’ compliance, its efficacy can be variable. If bedside manoeuvres fail to induce LVOTO > 50 mmHg, exercise stress echo is the most physiological instrument to evoke it.

Maximum provoked peak LVOTO ≥ 50 mmHg is considered the threshold for invasive treatment, in patients who have refractory symptoms despite a maximally tolerated dose of medical therapy, as it is the threshold where theoretical models examining the relationship between the gradient and stroke volume predict that obstruction becomes hemodynamically significant [43].

Transoesophageal echocardiography (TOE) has a role in patient selection for septal reduction therapies, determining the precise location of septal anatomy as well as the presence of coexisting mitral valve or papillary muscle abnormalities. Along with cardiac CT, it can help decide if surgical myectomy or alcohol septal ablation (ASA) should be pursued. TTE and/or TOE are essential pre-interventions to exclude aortic stenosis and subaortic membranes. Intraoperative TOE plays a key role in guiding the management of patients with HCM undergoing surgical myectomy and is critical for intraprocedural guidance of mitral valve transcatheter edge-to-edge repair (TEER) to treat patients with oHCM who are not candidates for septal reduction therapy [44].

For procedural planning of ASA, echocardiography contrast or agitated saline with radiographic contrast can be injected into septal arteries of interest. Myocardial contrast echocardiography plays a critical role in intraprocedural guidance of ASA. La Canna et al. demonstrated the value of myocardial contrast three-dimensional echocardiography to target safe and long-term effective septal reduction with ASA in patients with oHCM referred for isolated septal myectomy [45].

Postprocedural TOE assessment should assess if myectomy has been successful and examine for complications. TTE is used for post-procedural assessment following ASA. Some centres assess post-procedure LVOT gradients with dobutamine and/or isoproterenol [14].

### 4.3. Mitral Valve Assessment

Patients with oHCM often have abnormalities of the mitral valve and the subvalvular apparatus, which contribute to systolic anterior motion (SAM). Sherrid et al. report that drag, the pushing force of flow, rather than the previously hypothesized Venturi forces, is the dominant hydrodynamic mechanism for SAM [46]. Mitral regurgitation (MR) related to SAM of the mitral valve is typically posteriorly directed and in this case, septal myectomy alone will treat MR, without need for any valve intervention [12]. When the regurgitant jet is eccentric, quantitative assessment, e.g., the proximal isovelocity surface area (PISA) method, can lead to an erroneous estimation of MR severity. The presence of a central or anteriorly directed MR jet should prompt careful evaluation, including with TOE, as MR related to intrinsic mitral valve disease (e.g., mitral valve prolapse, chordal elongation, or rupture with flail) can often occur in patients with HCM and must be addressed separately [2,13]. If there is any concern over intrinsic mitral valve disease, TOE or CMR may enable a better evaluation of the mechanism of MR. LA enlargement provides important prognostic information and the most common mechanisms are SAM-related MR and elevated LV filling pressures [2].

### 4.4. Systolic Function

TTE provides information on global and regional LV and right ventricular anatomy and function. LVEF is only one measure of LV systolic performance when hypertrophy is present. Regional wall motion abnormalities can potentially be an early sign of disease. LVEF ranges from normal to hyperdynamic (55 to >70%), whereas an LVEF < 50% indicates LV systolic dysfunction and correlates with higher rates of adverse events, including all-cause mortality and cardiac transplantation [47]. Contrast can increase reliability of LVEF measurement, in particular, when imaging is suboptimal. Doppler myocardial velocities and deformation parameters (speckle tracking or tissue Doppler), i.e., global longitudinal strain, are more sensitive than LVEF in the detection of subtle ventricular dysfunction and may help discriminate between different aetiologies of hypertrophy (e.g., amyloidosis, HCM, Fabry disease, and athlete’s heart). LVEF assessment with three-dimensional (3D) TTE has been shown to correlate better with CMR compared to 2D, which typically underestimates it. It is believed that reduction in the global longitudinal strain (GLS) is counterbalanced by an increase in the global circumferential strain (GCS), resulting in a biplanar strain vector more circumferentially orientated and in a normal LVEF [13]. Strain abnormalities, in particular, regional abnormalities, vary according to the degree of LVH and could be pathological in segments even with relatively normal wall thickness [12]. These abnormalities are most likely due to underlying disarray or fibrosis and there is evidence that given the patchy distribution of fibrosis in HCM, a segmental analysis of the LV longitudinal strain may be even more accurate in assessing fibrosis than a global evaluation [13]. Wabich et al. demonstrated that the segmental longitudinal strain, rather than the GLS (with a cut-off value of −12.5%), has a higher sensitivity for the identification of LGE on CMR, and could enable better decision making of which patients require CMR for better risk stratification [48]. Although it is difficult to identify a prognostic cut-off value of the GLS, a reduction below −16% is an independent predictor for HF hospitalization, sustained ventricular arrhythmias, all-cause death, and atrial fibrillation occurrence [13].

### 4.5. Diastolic Function

Diastolic dysfunction is the hallmark of HCM and commonly, in symptomatic disease, there is evidence of impaired LV relaxation, as a consequence of increased myocardial stiffness, and impaired LA function. Although cardiac catheterization remains the gold standard to directly measure LV filling pressures, echocardiographic assessment of diastolic function provides a surrogate. As per ESC guidelines, routine examination should include mitral inflow assessment, tissue Doppler imaging, pulmonary vein flow velocities, pulmonary artery systolic pressure, and LA size/volume [2]. Exercise echocardiography provides important information regarding diastolic function. A restrictive LV filling pattern (E/A > 2, with increased E/e’ ratio > 14) in patients with HCM is associated with HF hospitalizations, reduced exercise tolerance, and SCD [13].

## 5. Cardiovascular Magnetic Resonance (CMR)

CMR plays an important role in the diagnosis, management, and risk stratification of patients with HCM. With its high spatial resolution, it can provide detailed assessment of anatomy, function, and tissue characterization.

### 5.1. Anatomy/Morphology and Function

CMR is a valuable modality to assess the thickness and distribution of LVH more accurately. CMR is the gold standard measure for cardiac function and is particularly helpful when echocardiographic acoustic windows are suboptimal especially in detecting LV apical and anterolateral hypertrophy [2] and in patients with abnormal electrocardiograms despite an apparently normal echocardiogram [2]. Machine learning measurement using MWT measurement with CMR in HCM has been shown to be superior to human experts with potential implications for diagnoses, risk stratification, and clinical trials [49].

CMR has a high sensitivity for LV apical hypertrophy, aneurysms, myocardial crypts, and papillary muscle abnormalities [50,51,52,53,54,55]. Apical HCM with an LV apical aneurysm is very rare (2.3% of all apical HCMs), often missed with echocardiography, and associated with an increased risk of adverse cardiovascular (CV) events compared with those with apical HCM but without an LV apical aneurysm [56].

### 5.2. Guiding Treatment/Procedures

CMR can be used to guide planning prior to surgical and catheter-based interventions (e.g., myectomy or alcohol septal ablation) in patients with HCM, especially if echocardiography acoustic windows are suboptimal, and in patients with multi-level or biventricular outflow obstruction [32]. CMR can also assess for mitral valve abnormalities (e.g., SAM) and LVOTO; however, this modality will usually underestimate the dynamic LVOT gradient compared to echocardiography. CMR helps identify anomalies of papillary muscles (e.g., insertion of anomalous, hypertrophied anterolateral papillary muscle directly into the anterior mitral leaflet (in the absence of chordae tendinae) represents an important mechanism of muscular midcavity obstruction), which help dictate specific surgical approaches [53].

### 5.3. Tissue Characterization (Multiparametric Mapping and Late Gadolinium Enhancement Imaging)—Differentiating HCM Phenocopies

**Figure 4:**
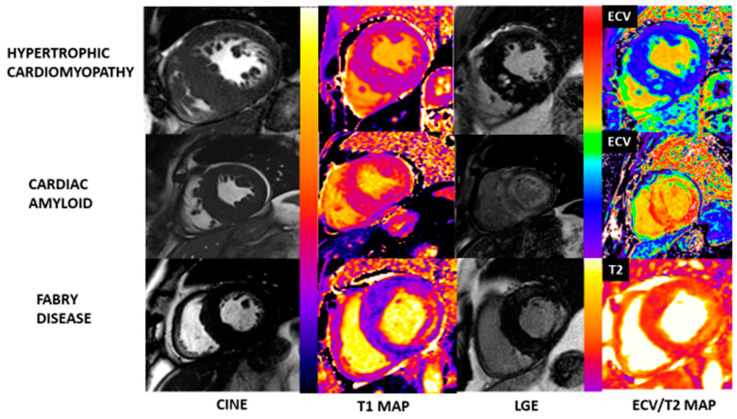
Hypertrophic cardiomyopathy and HCM phenocopies with tissue characterization assessment using CMR. In HCM, native T1 and ECV can be either normal or mildly elevated with patchy midwall LGE especially seen in hypertrophic regions. LGE at RV insertion points can also be present. In cardiac amyloidosis, native T1 and ECV is significantly elevated with either subendocardial or transmural LGE throughout the myocardium. In Fabry disease, native T1 is typically low with elevated T2 at corresponding LGE areas with typical basal inferolateral LGE.

CMR can differentiate phenocopies of HCM using multiparametric mapping. Fabry disease is typically associated with low native T1 values compared with patients with HCM and left ventricular hypertrophy (LVH) and approximately 40–59% of patients who are LVH-negative [57,58]. Fabry disease is also typically associated with basal inferolateral LGE and most present with concentric LVH [57,59]. Cardiac amyloid is typically associated with high native T1 and extracellular volume fraction (ECV) values, abnormal myocardial nulling, and diffuse subendocardial LGE (Figure 4) [2,60]. CMR is also useful in aiding the diagnosis for athlete’s heart and hypertensive heart disease. ECV is found to be low in athlete’s heart compared to healthy volunteers. In athlete’s heart, as LVH increases, ECV decreases. However, in patients with HCM, as LVH increases, ECV also increases, suggesting that the increase in left ventricular mass (LVM) in HCM is mediated by cellular disarray and extracellular matrix expansion [61].

In HCM, T2 values at the area of LGE are found to be elevated compared to healthy volunteers; however, T2 values at the area of LGE are significantly more elevated in Fabry disease compared to patients with HCM [62,63]. Some patients with HCM had focal T2 abnormalities that matched areas of LGE, and these abnormalities were associated with severe LVH [64].

### 5.4. LGE as Risk Stratification

Myocardial biopsy is infrequently performed and thus CMR provides a non-invasive tool to assess myocardial fibrosis in patients with HCM [65]. When interstitial fibrosis is present, the ECV and T1 times of the myocardium are increased [65]. Myocardial fibrosis has prognostic value in patients with HCM.

The presence and extent of LGE, a measure for myocardial fibrosis, have prognostic value in predicting adverse CV events (SCD, CV death, HF death, all-cause death) in patients with HCM [66,67]. In patients with HCM, LGE weakly correlated with hypertrophy, was inhomogeneous and asymmetric, and predominantly distributed in the interventricular septal wall and anterior free wall at basal and mid-levels [68]. A greater extent of LGE is associated with a poor prognosis, regardless of its location in the LV [68]. Extensive LGE provides additional information to assess SCD risk in patients with HCM, particularly in those otherwise judged to be at low risk [69]. LGE has also been associated with a greater likelihood and increased frequency of ventricular tachyarrhythmias including NSVT [70].

There is a need to consider the standardization of different LGE quantification techniques, even though they have comparable accuracy in predicting SCD in patients with HCM. The most common technique is the 6 SD technique. Less common techniques include manual quantification, the 4 SD technique, and the 2 SD technique [70,71].

Myocardial fibrosis (LGE) is progressive in some patients with HCM, with impaired energetics and perfusion abnormalities postulated as possible mechanistic drivers of the fibrotic process [72]. The typical perfusion abnormality in HCM is small vessel ischemia (coronary microvascular dysfunction), which in time leads to fibrosis, although some postulate that the remodelling of the microcirculation at the arteriolar and capillary levels might also occur secondary to the fibrosis [73,74]. Moreover, there is growing evidence for the activation of fibrotic pathways occurring early in the course of the disease before hypertrophic remodelling and microvascular dysfunction and ischemia occur [75]. Fibrosis progression is associated with adverse cardiac remodelling and predicts an increased risk of subsequent clinical events in HCM [72].

Myocardial LGE radiomics (i.e., shape and texture features) have been shown to be strongly associated with SCD risk in HCM, providing incremental risk stratification beyond current ESC or AHA/ACC risk models [76]. The ESC guidelines recommend that the presence of extensive LGE (≥15%) may be used as part of shared decision making with patients about prophylactic ICD implantation for low to intermediate risk patients [2]. It has been suggested that the addition of LGE to the current AHA/ACC sudden death algorithm [31] or the HCM-SCD risk model improves stratification but there is scant robust data on the impact of scar quantification on the personalized risk estimates generated with the HCM-SCD risk calculators [2].

The Hypertrophic Cardiomyopathy Registry (HCMR) is the largest CMR and genetics international prospective registry in HCM with *n* = 2755 patients recruited [21]. HCMR is designed to assess the role of CMR in risk stratification in HCM, and also incorporates genetic and biomarker data. In HCMR, sarcomere variant (+) patients were more likely to have reverse septal curvature morphology, LGE, and no significant resting LVOTO. On the other hand, sarcomere variant (−) patients were more likely to have isolated basal septal hypertrophy, less LGE, and more LVOTO. Interstitial fibrosis (measured with ECV), was present in segments both with and without LGE. Of note, serum N-terminal pro-B-type natriuretic peptide (NT-proBNP) and cardiac troponin T levels correlated with increasing LGE and ECV.

### 5.5. CMR Perfusion and Microvascular Dysfunction

CMR perfusion can detect myocardial perfusion abnormalities in patients with HCM, felt to reflect microvascular dysfunction, which is common in patients with HCM, and associate with hypertrophy and LGE. Apical perfusion defects are found to be universally present in apical HCM at all stages alongside characteristic ECG changes, suggesting that ischemia may play a disease-defining role in apical HCM [77].

First-pass perfusion CMR identifies abnormal rest perfusion in a significant proportion of patients with HCM, with abnormalities associated with the presence and distribution of a myocardial scar and the degree of hypertrophy [78]. Rest perfusion abnormalities identify patients with an increased incidence of episodes of NSVT, independently from the presence of myocardial fibrosis [78]. The regional heterogeneity of resting perfusion in HCM is related to delayed contrast enhancement but not to systolic function [79].

In 101 patients with HCM with unobstructed coronaries using quantitative myocardial stress perfusion imaging, global stress myocardial blood flow (MBF) and myocardial perfusion reserve (MPR) were lower in HCM than controls. Stress myocardial blood flow (MBF) fell with increased LV mass, MWT, and LGE (*p* < 0.0001). Normal segments (no LVH/LGE) had reduced stress MBF and MPR compared to controls, suggesting it may be an early disease marker [80]. In patients with HCM, adenosine-stress perfusion defects on CMR were found in >40% of subjects; these perfusion defects were associated with NSVT, higher indexed LVM (LVMi), and apical aneurysms [81].

### 5.6. Diffusion Imaging

Cardiac diffusion tensor imaging (cDTI) allows the in vivo characterization of myocardial microstructure by quantifying the mean diffusivity (MD), fractional anisotropy (FA) of diffusion, and secondary eigenvector angle (E2A) [82,83]. Patients with HCM showed reduced mobility with altered diastolic conformation [83]. In patients with HCM, even in segments with normal wall thickness, normal perfusion, and no scar, diffusion is more isotropic than in controls, suggesting the presence of underlying cardiomyocyte disarray. Increased E2A suggests the myocardial sheetlets adopt hypercontracted angulation in systole. Increased MD, most notably in the subendocardium, is suggestive of regional remodelling, which may explain the reduced subendocardial blood flow. These findings provide a greater understanding of HCM pathophysiology.

Microstructural alteration using cardiac diffusion tensor imaging and microvascular dysfunction using quantitative perfusion CMR can occur in the absence of hypertrophy in sarcomeric gene variant carriers, in whom changes are associated with ECG abnormalities. This is potentially important in the emerging era of disease-modifying therapy in HCM [84].

### 5.7. Strain

CMR feature tracking reveals LV and LA dysfunction in patients with HCM, even amongst those with normal LVEF [85]. An impaired LV strain has been associated with elevated NT-proBNP and/or high-sensitivity cardiac troponin T in patients with HCM [85]. The CMR tissue tracking (CMR-TT)-derived LV global longitudinal peak diastolic strain rate (PDSR) can predict adverse outcomes in patients with HCM—those with lower longitudinal PDSR had lower freedom from major adverse cardiovascular events (MACEs) [86]. The combination of maximum LVWT and the subradial peak strain is independently associated with LVOTO in patients with HCM [87]. GCS (with CMR-TT) is an independent risk indicator of ventricular arrhythmias in HCM [88].

CMR feature tracking, especially the regional circumferential strain, was associated with (LGE) fibrosis-containing segments in HCM [89]. In patients with HCM, the 2D peak segmental longitudinal strain is an excellent strain parameter for tissue characterization and fibrosis detection [90]. However, 2D and 3D deformation parameters are not interchangeable, showing only modest correlations [90].

MRI tagging has also been used to help confirm the presence of contractile function in a suspected mass, for example, in the case of focal HCM simulating a mass [91]. Fast 3-breathhold 3D tagging is feasible for regional and global strain analyses in patients with HCM [92]. Following alcohol septal ablation in patients with symptomatic oHCM, the reduction of LVOTO was associated with improved intramural systolic function (CMR tagging and 3D strain), indicating reverse LV remodelling [93].

### 5.8. Flow

The quantification and visualization of elevated velocity in the LV is feasible in patients with HCM, providing insights into altered hemodynamics [94]. Four-dimensional flow CMR offers a new way to assess intraventricular diastolic flow and non-invasively evaluate myocardial stiffness. A study integrated 4D-flow and T1-mapping analyses in HCM, and findings suggest a mechanistic link between abnormal LVOT flow, increased LV loading, and adverse myocardial remodelling in HCM [95].

## 6. Cardiac Computed Tomography (CT)

Cardiac CT allows the non-invasive evaluation of epicardial coronary artery disease in patients with HCM. CT is helpful as an adjunct or alternative to other imaging modalities, e.g., if TTE is non-diagnostic or CMR is not feasible.

### 6.1. Anatomy/Morphology

CT imaging provides high-resolution images that allow detailed assessment of patterns and distribution of myocardial hypertrophy, and ventricular size.

### 6.2. Function

Biventricular volume and systolic function quantification is feasible with the new-generation CT scanners, from dual-source scanners to wide detectors [96]. These enable the entire cardiac volume to be covered in one heartbeat. Reduction in gantry rotation time is associated with increased temporal resolution and improves end-systolic and end-diastolic phase identification. However, the routine clinical use of cardiac CT for volumes and function is debatable due to the requirement for a dedicated acquisition protocol with an elevated radiation dose and higher doses of the contrast medium.

### 6.3. Epicardial Coronary Artery Disease (CAD)

Contrast-enhanced, ECG-gated cardiac CT is an effective non-invasive modality to evaluate for obstructive epicardial CAD, especially for those with a low-to-intermediate probability of CAD (15–50%) [12]. Obstructive epicardial CAD is reported to be present in 7–19% of patients with HCM [97,98,99]. Importantly, cardiac CT coronary angiography (CTCA) tends to overestimate severity compared to invasive angiography [12]. Associated coronary artery anomalies can also be detected.

Patients with HCM and chest pain have been found to have a lower prevalence of moderate to severe epicardial coronary artery stenosis on CTCA, compared with the risk-adjusted general population [97]. Given the high incidence of false-positive findings in perfusion stress studies, CTCA can be useful to triage for coronary angiography in patients with HCM and angina [98].

### 6.4. Three-Dimensional Reconstruction and Pre-Procedural Planning

Advanced post-processing algorithms allow for the creation of 3D reconstructions of the heart and epicardial coronary arteries, which can aid in the understanding of complex anatomical relationships and provide a roadmap to guide surgical or catheter-based interventions. For example, in planning alcohol septal ablation for oHCM, CTCA can help identify the target vessel for the optimum infarct location [100]. Cardiac CT can provide important insights on septal anatomy for a safer and more effective procedure [101].

### 6.5. Anomalous Coronary Anatomy and Myocardial Bridges

CTCA is the reference standard for imaging anomalous coronary anatomy and myocardial bridges. Myocardial bridging may cause the compression of an epicardial coronary artery. Myocardial bridges were seen in as many as 40.7% of patients with HCM [97]. Myocardial bridges in patients with HCM are reported to be longer and deeper, when compared to a control group [97]. The role of myocardial bridges and their association with sudden death are unclear in patients with HCM [102].

### 6.6. CT-Based Fractional Flow Reserve (CT-FFR)

Whilst CTCA allows anatomical assessment of coronary lesions, CT-FFR also allows non-invasive functional assessment of intermediate-severity coronary lesions. Patients with HCM have slightly lower CT-FFR values in the distal vessels, even in the absence of severe CAD—this may be due to a disproportionate increase in demand (myocardial mass) vs. supply (coronary capacity) [103]. In patients with a larger LVM, there is an increased discrepancy between CT-FFR and invasive FFR values [104]. CT-FFR also allows the evaluation of the coronary artery volume to myocardial mass ratio (V/M). Patients with HCM have been shown to demonstrate a significantly greater coronary volume, yet have decreased V/M [103].

### 6.7. Dual-Energy Cardiac CT and Tissue Characterization (Late Iodine Enhancement, ECV)

Dual-energy cardiac CT, a newer form of imaging, can provide additional data on tissue characterization. It allows the assessment of late iodine enhancement and ECV quantification, with results comparable to CMR [105]. However, larger prospective studies are required before this technique can be routinely applied in clinical practice [106]. Further refinement of the contrast infusion protocol and imaging parameters is required before it can be used routinely [107]. One case report has reported the utility of dual-energy CT delayed myocardial enhancement to help differentiate between a true vs. false LV aneurysm [108].

### 6.8. Limitations: Radiation, Contrast, Vasodilators, and Image Optimization

Whilst radiation exposure is a limitation of CT, new CT technology and good heart rate modulation can allow for lower doses of radiation exposure. An iodine contrast medium is required, but not advised in those with reduced eGFR < 30 mL/min. Although vasodilators such as nitroglycerin are helpful during cardiac CT imaging, these should be avoided when severe LVOTO is present. Image quality is best in patients with a low heart rate and regular rhythm.

In patients less suited for CTCA—e.g., due to safety concerns (radiation, iodine contrast) or arrhythmia—functional tests (e.g., MRI perfusion or quantitative PET) may be more appropriate to evaluate for coronary artery disease in HCM.

Technological advancements in CT have improved resolution, enabled functional assessment and tissue characterization, and reduced radiation exposure whilst reducing scan times, thus enhancing its role in HCM.

## 7. Nuclear Imaging

HCM can lead to inadequate blood supply to the myocardium due to microvascular dysfunction or mechanical obstruction. Nuclear imaging includes myocardial perfusion imaging (MPI) techniques like single-photon-emission computed tomography (SPECT) and positron emission tomography (PET), which can define the presence and severity of ischemia in HCM. PET can also evaluate MBF and the flow reserve. Nuclear imaging is generally not needed to evaluate anatomy (e.g., wall thickness) due to its low spatial resolution and radiation exposure, in contrast with TTE and CMR, which offer high spatial resolution without radiation exposure [12].

### 7.1. SPECT vs. PET and Radiotracers

The preferred SPECT radiotracer is technetium-99 m due to a short half-life (6 h vs. 73 h for thallium-201) and lower radiation exposure [12]. Due to the long half-lives of both radiotracers, SPECT stress images are acquired 15–60 min after peak stress and maximum hyperaemia.

PET MPI is superior to conventional SPECT because of its precise built-in attenuation correction, resulting in improved image quality, allowing for an accurate quantitative analysis of MBF. PET scan durations are shorter due to the relatively shorter half-lives of the radiotracers (rubidium-82: 75 s and N-13 ammonia: 10 min) [12]. PET allows a more accurate quantification of the stress ejection fraction, transient ischemic dilatation, and MBF reserve [109].

### 7.2. Stress—Patterns

Stress with either exercise or vasodilators is frequently used and typically demonstrates one of three patterns: (1) normal perfusion; (2) a reversible perfusion defect in the area of the greatest hypertrophy; (3) diffuse subendocardial ischemia from microvascular disease, leading to ischemic dilation of the LV and a decrease in LVEF [12]. Pattern number 3 is typically seen with concentric HCM, whilst focal areas of hypertrophy may show reversible perfusion defects [12]. Approximately half of patients with HCM have an abnormal ejection fraction response to stress [110]. Some show transient ischemic dilatation [111].

### 7.3. Ischemia—Prevalence, Patterns, and Prognosis

MPI can detect areas of ischemia that may not be apparent on other imaging modalities. Perfusion defects can be detected in patients with HCM even without significant epicardial coronary artery disease. One study found a high prevalence of thallium perfusion abnormalities in 39% of patients with HCM despite normal epicardial coronary arteries [112]. Due to a higher uptake in hypertrophic segments, relatively abnormal perfusion can be found in non-hypertrophied regions [113]. The presence and extent of perfusion defects on MPI are associated with an increased risk of adverse cardiac events (e.g., cardiac arrest, syncope) in HCM [114]. In patients with abnormal MPI tests, evaluation for epicardial CAD should be considered.

### 7.4. Genotype-Positive

Patients with HCM with sarcomere myofilament gene variants have more severe impairment of microvascular function, assessed with dipyridamole MBF using 13N-labelled ammonia [115].

### 7.5. PET MBF and Prognosis

Resting MBF is typically normal in patients with HCM. However, areas with a significant scar may have decreased MBF [116]. The blunted augmentation of MBF during stress compared with rest might be seen either globally or in the areas of greatest hypertrophy [12]. An abnormal MBF reserve may be seen in both hypertrophied and non-hypertrophied areas [117]. MWT is a strong predictor of an impaired MBF reserve [118]. The MBF reserve is prognostically important, predicting clinical deterioration and death in patients with HCM [119].

### 7.6. Assessing Therapeutic Efficacy

Nuclear imaging can help evaluate treatment efficacy. For example, CCBs and surgical myectomy can improve perfusion patterns in patients with HCM [120,121].

### 7.7. Hybrid Imaging Techniques

The combination of nuclear imaging with CT (e.g., PET/CT or SPECT/CT) and MRI (e.g., PET/MRI) allows for the simultaneous assessment of anatomy/structure and function/physiology, providing a more comprehensive evaluation [122,123]. For example, myocardial fibrosis has been demonstrated with integrated cardiac F-18 FDG PET/MR in patients with HCM [124].

## 8. Role of MMI in Clinical Research and Trials

Multi-modality imaging plays an important role in clinical research to understand disease biology and as a surrogate efficacy endpoint in clinical trials (Table 2 and Table 3).

### 8.1. Endpoint Determination (Efficacy, Safety)

Imaging endpoints, such as changes in LV wall thickness, LV volumes, LA volumes, and fibrosis, are often used in trials to assess the efficacy of therapeutic interventions. In trials of cardiac myosin inhibitors (CMIs), TTE plays a crucial role in assessing change in LVOT gradients (efficacy) and LVEF change (safety), which guide dose titration.

### 8.2. Confirm Diagnosis and Determine Eligibility

Detailed imaging can help in selecting the right patient cohort by confirming the diagnosis and disease type, e.g., obstructive versus non-obstructive HCM.

### 8.3. Mechanism of Action

Imaging can shed light on how therapies impact myocardial structure and function. For example, the CMI mavacamten has been shown to cause beneficial cardiac remodelling through reduction in the LVM index and maximum LA volume index [25].

## 9. Challenges and Opportunities

### 9.1. Standardization of Imaging Protocols and Analysis Technique

Standardization is critical in clinical research and trials to ensure that imaging data are consistent and comparable across different sites and over time. This involves implementing uniform protocols (acquisition and post-processing), training, and quality control.

### 9.2. Tailored Imaging Strategy

Given the diverse manifestations of HCM, an individualized approach to imaging is often necessary. Imaging modalities can be selected based on the patient’s risk profile, symptoms, and stage of the disease. It is also important to consider other patient-specific factors, such as age, gender, BSA, and the presence of comorbid conditions, in the interpretation of imaging findings for diagnoses and tailoring treatment as part of precision medicine. Other things to consider are contraindications to certain imaging modalities (e.g., advanced renal failure may contraindicate the use of gadolinium contrast in MRI). Table 4 summarizes the strengths and limitations of cardiac imaging modalities in HCM.

### 9.3. Heterogeneity of Phenotypes

Imaging strategies may differ for various subtypes of HCM, such as obstructive vs. non-obstructive, apical hypertrophy, or the presence of apical aneurysms. Imaging can help elucidate the underlying pathophysiology, whether it is predominantly hypertrophy, fibrosis, or myocardial disarray.

### 9.4. Timing of Imaging

The recommended frequency of follow-up cardiac imaging in patients with HCM varies based on several factors, including the patient’s symptoms, stage of disease, risk profile, treatment regimen, and changes in clinical status. These decisions are best made in conjunction with a cardiologist who specializes in HCM. Individuals with a higher risk profile (e.g., severe LVH, history of NSVT, abnormal blood pressure response to exercise, family history of SCD, LGE) and genetic predispositions may need more frequent imaging.

### 9.5. Predictive Modelling

Advanced imaging data can feed into predictive models to improve risk stratification and patient counselling.

## 10. Conclusions

In summary, multi-modality imaging in HCM provides comprehensive insights into the anatomical, functional, and prognostic aspects of the disease. This improves our understanding, allows for better patient stratification, and helps in evaluating therapeutic interventions as emerging novel disease-modifying treatments are being developed. As imaging technology advances, its role in HCM research is likely to grow, offering opportunities for more personalized and effective treatments.

## Figures and Tables

**Table 1:** ESC diagnostic criteria of hypertrophic cardiomyopathy [2].

Adults	LV wall thickness ≥ 15 mm in any myocardial segment that is not explained solely by loading conditions. LV wall thickness of 13–14 mm requires evaluation of family history, genetic findings, and ECG abnormalities.
Children	LV wall thickness z-score > 2.
Relatives	LV wall thickness ≥ 13 mm. In child first-degree relatives with LV wall thickness z-scores of <2, the presence of associated morphological or ECG abnormalities should raise the suspicion but are not diagnostic for HCM.

ECG: electrocardiogram, HCM: hypertrophic cardiomyopathy, LV: left ventricular, z-score: number of standard deviations from predicted mean.

**Table 2:** Randomized placebo-controlled trials with imaging outcomes.

Treatment	Trial/Year/N/Duration	Outcomes
Exercise	RESET-HCM (2017) [125] NCT01127061 N = 136 randomized N = 47 to 61 CMR follow-up data samples 16 weeks	Echo (secondary): ↔ LVOT-G (Valsalva/exe) CMR (secondary): ↔ Maximal LV thickness ↔ LVMi ↔ LVEDVi ↔ LVESVi ↔ LVEF ↔ Total DGE ↔ DGE % of LV mass
Losartan	NCT01150461 (2013) [126] N = 20 1 year	CMR (primary): ↓ % LGE CMR (secondary): ↔ LVM
N-Acetylcysteine	HALT-HCM (2018) [127] NCT01537926 N = 35, echo (per-protocol analysis); N = 18, CMR (per-protocol analysis) 12 months	Echo (secondary): ↔ LVESD ↔ LVMi ↔ LVM CMR (secondary): ↔ MWT ↔ LVEDV ↔ LVESV ↔ LVEF ↔ Mean LV midwall strain ↔ Myocardial mass ↔ Enhanced myocardium ↔ % of myocardium that has scar
Valsartan	VANISH (2021) [128] NCT01912534 N = 178 2 years	Echo (secondary): ↑ E’ velocity ↔ S’ velocity CMR (secondary): ↔ Max LV wall thickness ↔ LVMi ↔ LAVi ↑ LVEDV ↔ LVESV
Mavacamten	EXPLORER-HCM (2021) [23,25] NCT03470545 N = 251 N = 35, CMR substudy Week 30	Echo (secondary): ↓ LVOT-G (exercise) CMR (exploratory): ↓ LVMi ↓ Max LV wall thickness ↓ LAVI max ↓ LVEF ↔ LGE
	EXPLORER-CN (2023) [129] NCT05174416 N = 81 (including N = 58 CMR data samples) Week 30	Echo (primary): ↓ LVOT-G (Valsalva) Echo (secondary): ↓ LVOT-G (rest) ↓ Proportion of LVOT-G < 30 and <50 CMR (secondary): ↓ LVMi CMR (exploratory): ↓ LVM and ↓ LV MWT ↓ Max LAVi and ↓ min LAVi
Aficamten	SEQUOIA-HCM (ongoing) [130] NCT05186818 N = 282 (includes CMR substudy) 12/24 weeks	Echo (secondary) (12/24 weeks): LVOT-G (Valsalva) Proportion of LVOT-G < 30 Echo (safety): Incidence of LVEF < 50% Echo (exploratory) (24 weeks): LVEF LVESV, LVEDV LAV CMR (exploratory) (24 weeks): LVMi LVEF Septal, free wall, MWT LAVi LVESV LVEDV
	FOREST-HCM (ongoing) [131] NCT04848506 N = ? (CMR substudy) Up to 5 years	Echo (secondary) (12-week intervals): Peak LVOT-G at rest
Perhexilene	RESOLVE-HCM (ongoing) [132] NCT04426578 N~60 12 months	CMR (primary): LVH (septal thickness) CMR (secondary): LVM Oxygen-sensitive CMR
Trientine	TEMPEST (ongoing) [133] NCT04706429 N = 154 Week 52	CMR (primary): LVM/BSA CMR (secondary): LV GLS and strain rate Wall thickness, mass, volumes, EF Atrial volume and function CMR (mechanistic): LV myocardial cellular mass, LV myocardial extracellular mass, myocardial ECV, LV LGE PCr/ATP ratio (31P MRS) (subgroup)
Moderate-intensity exercise training vs. usual physical activity	EXCITE-HCM [134] NCT05818605 (ongoing) N~70 24 weeks	Echo (secondary): Myocardial systolic strain Myocardial work PET (exploratory): Regional myocardial perfusion Coronary flow reserve (ratio)

↑: Increased, ↓: Decreased, ↔: Unchanged, CMR: cardiac magnetic resonance, CT: computed tomography, ECV: extracellular volume fraction, EF: ejection fraction, GLS: global longitudinal strain, LAV: left atrial volume, LAVi: left atrial volume index, LGE: late gadolinium enhancement, LV: left ventricular, LVEDV: left ventricular end diastolic volume, LVEDVi: left ventricular end diastolic volume index, LVEF: left ventricular ejection fraction, LVESV: left ventricular end systolic volume, LVESVi: left ventricular end systolic volume index, LVH: left ventricular hypertrophy, LVM: left ventricular mass, LVM/BSA: left ventricular mass/body surface area, LVMi: left ventricular mass index, LVOT: left ventricular outflow tract, LVOT-G: left ventricular outflow tract gradient, MWT: maximal wall thickness, PCr/ATP: phosphocreatine/ATP ratio, PET: positron emission tomography.

**Table 3:** Non-randomized treatment trials with imaging outcomes.

Treatment	Trial/Year/N/Duration	Outcomes
Ranolazine	NCT03953989 [135] N = 26 4 months	PET (primary): MBF during hyperaemia Coronary flow reserve Coronary resistance
Non-Invasive Radiation Ablation	NIRA-HOCM [136] NCT04153162 N~10 3/6/12 months	CT (12 months) (secondary): Patency of LAD artery Presence of radiation pneumonitis Echo (3/6/12 months) (secondary): Aortic and mitral valve function LVOT-G LVEF CMR (6 months) (secondary): LV wall thickness
Exercise	NCT04580693 [137] N~60 2 weeks 3 groups: endurance athletes, HCM, healthy volunteers/control	PET (secondary): MBF reserve Echo (exploratory): LVM
Transcatheter Intra-septal RF Ablation System (TIRA Catheter)	First-in-Man early Feasibility Study for Transcatheter HOCM Septal Ablation [138] NCT04770142 N~7 1 month	Echo (primary): LVOT-G (rest/Valsalva) LVOT diameter IVS CT and MRI (primary): IVS
Renal Denervation	SNYPER-PS (ongoing) [139] NCT05577208 N~20 6 months	SPECT (primary): Cardiac sympathetic nerve activity (123I-MIBG washout rate measured with scintigraphy) Echo (secondary): LVM LVOT-G (Valsalva)
Mavacamten	MavaPET (ongoing) [140] NCT06023186 N~20, oHCM 12 months	PET-CT (primary): Myocardial perfusion reserve

CMR: cardiac magnetic resonance, CT: computed tomography, IVS: intraventricular septum, LAD: left anterior descending, LV: left ventricular, LVEF: left ventricular ejection fraction, LVM: left ventricular mass, LVOT: left ventricular outflow tract, LVOT-G: left ventricular outflow tract gradient, MBF: myocardial blood flow, MRI: magnetic resonance imaging, PET: positron emission tomography, PET-CT: positron emission tomography–computed tomography, SPECT: single-photon emission computed tomography.

**Table 4:** Strengths and limitations of cardiac imaging modalities in HCM.

Imaging Modality	Strengths	Limitations
Echocardiography	Relatively inexpensive Widely available Good assessment of wall thickness, chamber size, and systolic/diastolic function Good assessment of dynamic LVOT and mid-cavity gradients Excellent valvular assessment No radiation Few contraindications TTE and TOE can be used perioperatively to aid septal reduction therapy	Operator variability Reliance of good acoustic windows
Cardiac magnetic resonance	Gold standard for wall thickness, chamber size, and systolic function Use of gadolinium contrast, allows identification of fibrosis/scarring (presence of LGE) Excellent for tissue characterisation to exclude HCM phenocopies Good valvular assessment Excellent myocardial perfusion assessment No radiation	Relatively expensive Less widely available Claustrophobia Limitations if ferromagnetic implant or devices are present in some CMR centres Limitation if there is advanced renal dysfunction (eGFR < 30 mL/min) Tend to underestimate LVOT gradient
Cardiac computed tomography	Excellent assessment of surrounding anatomical structures Excellent coronary artery disease and anatomy assessment Reasonable assessment of wall thickness and chamber size	Less widely available Use of radiation Limitations if there is renal dysfunction (eGFR < 30 mL/min)
Nuclear imaging	Excellent myocardial perfusion assessment Can be used to aid diagnosis of ATTR cardiac amyloidosis (HCM phenocopy)	Less widely available

ATTR: Transthyretin amyloidosis, CMR: cardiac magnetic resonance, eGFR: estimated glomerular filtration rate, HCM: hypertrophic cardiomyopathy, LGE: late gadolinium enhancement, LVOT: left ventricular outflow tract, TTE: transthoracic echocardiogram, TOE: transoesophageal echocardiogram.
